# Topical Probiotics Reduce Atopic Dermatitis Severity: A Systematic Review and Meta-Analysis of Double-Blind, Randomized, Placebo-Controlled Trials

**DOI:** 10.7759/cureus.70001

**Published:** 2024-09-23

**Authors:** Elliot Flint, Nabeel Ahmad, Kevin Rowland, Charles Hildebolt, David Raskin

**Affiliations:** 1 Biomedical Sciences, University of Houston Tilman J. Fertitta Family College of Medicine, Houston, USA; 2 Anthropology, Washington University in St. Louis, St. Louis, USA

**Keywords:** atopic dermatitis, eczema, microbiota, probiotics, scorad

## Abstract

Atopic dermatitis (AD) is a chronic skin disease that commonly appears during childhood but can present at any age. There are interventions that are effective in treating AD in children, but it has been difficult to find effective treatments for adults. This systematic review and meta-analysis sought to determine the efficacy of topical probiotic treatment for AD in adult populations. A database search was conducted of peer-reviewed, double-blind clinical trials, and studies underwent a systematic exclusion and inclusion process, yielding four that met the criteria. Disease severity, as measured by a standardized scoring tool SCORAD (SCORing Atopic Dermatitis), was culled from each study and compared to placebo at two-week and four-week time points. All studies showed improvement in SCORAD in the treatment groups compared to baseline at all time points. Two showed significant decreases in SCORAD after two weeks of treatment, and three studies showed long-lasting improvement after four weeks of treatment. Interestingly, while each study showed a reduction in the severity of AD at the two- and four-week time points, a pooled meta-analysis did not show a statistically significant difference between treatment and control at four weeks of treatment. Clinically, there may be benefits to topical probiotic usage as evidenced by the individual studies; more studies need to be performed including adults to show statistical significance.

## Introduction and background

Atopic dermatitis (AD) is an inflammatory skin disorder that often appears in early childhood, affecting approximately 8-12% of children [1]. AD can become a chronic condition, extending into adulthood, with 6-9% of US adults affected [1]. Globally, more than 220 million people currently live with AD, with up to 10% of all adults being affected [2]. AD produces dry, itchy, and inflamed skin. While the etiology of AD is not fully understood, there are several factors known to be involved. Disruptions to the integrity of the epithelial layer of the skin are involved in AD. The filaggrin (FLG) protein helps maintain the integrity of the skin, and mutations in the FLG gene that produce loss of FLG function have been noted in AD [3]. Family history plays a clear role, as do environmental triggers, such as dust mites, heat, or a dry and humid climate [3]. Immune dysregulation plays a role in AD. In the innate immune system, there is reduced Toll-like receptor (TLR) function in antigen-presenting cells which has been found in patients with AD, leading to altered inflammatory response and changes in the skin microbiome [4]. In AD, the adaptive immune system produces modified cytokine signaling leading to the thickening of the epidermis. The skin of AD lesions is characterized by microbial dysbiosis, with a reduction of diversity and overrepresentation of *Staphylococcus aureus*, correlating with increased lesion infection and flare-ups [5]. The normal microbiomes of the gut and the skin are likely to play a role in AD development. Components of the microbiome produce molecules that inhibit the growth of *S. aureus*. Additionally, the microbiome regulates inflammatory responses that affect skin integrity and prevent the growth of pathogens [6,7]. Damage to the skin barrier allows pathogens to reach deeper layers of skin and produce inflammation.

Probiotics are living microorganisms that provide health benefits when consumed or applied to the body. Probiotics have been shown to modulate the immune system and prevent the growth of pathogens. Given the relationship between the gut and skin microbiomes and AD, many studies have been performed testing whether probiotics could relieve AD symptoms. Several systematic reviews reported the effects of oral probiotics and their effect on decreasing AD severity in adult and pediatric populations. Such reviews explored various oral probiotic strains of *Bifidobacterium*, *Lactobacillus*, and/or *Streptococcus *with most trials concluding a reduction in the SCORAD (SCORing Atopic Dermatitis) index [8-14]. SCORAD is a measure of the area and intensity of eczema lesions, along with a measure of itchiness and sleepiness of patients [3].

There is a growing body of research investigating the effectiveness of probiotics in reducing the severity of AD. Most of these studies use oral probiotics and many test effectiveness only in children. There have been fewer studies using topical probiotics and testing their effectiveness in adults. Thus, a systematic review of randomized controlled trials of topical probiotics is needed to establish the validity of existing research regarding treating AD. To date, there has been no systematic review of topical probiotics for AD. In this systematic review, we explored the literature on topical probiotics' role in decreasing AD in adult and pediatric populations. We performed a meta-analysis of the reported data to evaluate the effectiveness of the topical probiotics in reducing AD severity.

Data from this article was previously presented as a meeting abstract at the 2024 American Society for Microbiology Microbe meeting on June 16, 2024, and as a meeting abstract at the 2024 American Academy of Dermatology Annual Meeting in San Diego on March 8, 2024. This article was previously posted to the medRxiv preprint server on July 30, 2024.

## Review

Materials and methods

Study Design

**Figure 1:**
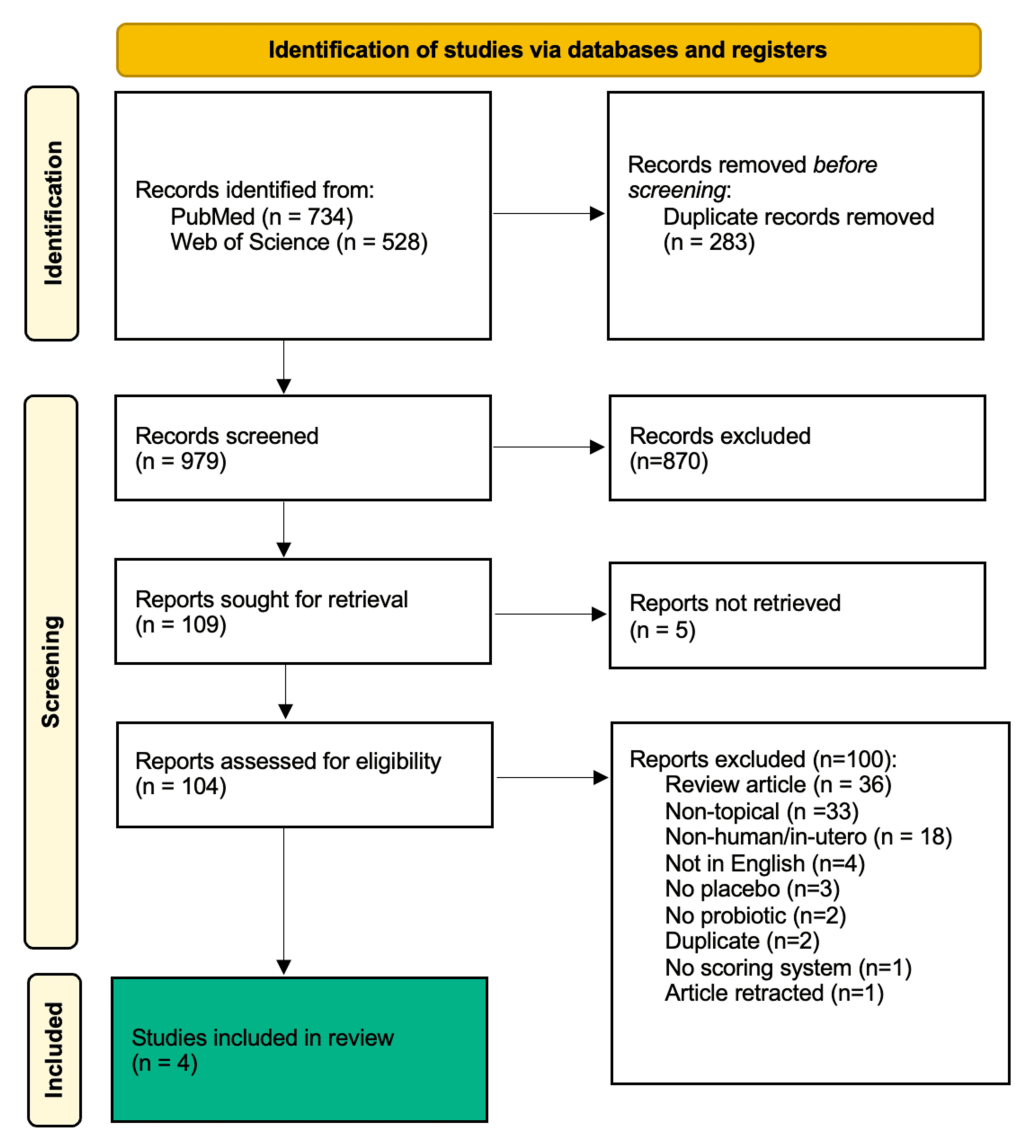
PRISMA flow diagram PRISMA: Preferred Reporting Items for Systematic Reviews and Meta-Analyses

The Web of Science and PubMed were searched using the terms (atopic dermatitis OR eczema) AND (probiotic* OR synbiotic*). After removing duplicates, the titles and abstracts of 979 articles were screened by two reviewers (EF and NA). Reviewers voted to exclude articles or advance them to the next stage of the review using the inclusion and exclusion criteria listed in Table 1. Reviewers' decisions were blinded to one another and organized by the systematic review software Rayyan (Qatar Computing Research Institute, Ar-Rayyan, Qatar). Any article that did not receive two votes to "exclude" was advanced to the next stage of the screening process (n=80). Article retrieval and screening of the full text of studies was done by four reviewers (EF, NA, DR, and KR). Each article was reviewed by two reviewers and voted to include or exclude based on the eligibility criteria. Conflicts between reviewer decisions were resolved by a group consensus of the four reviewers. At the end of the screening process, only one article met the eligibility criteria for inclusion in the review. To expand the pool of eligible studies, the reviewers eliminated "Study subjects of 18 years or older" as part of the eligibility criteria. Articles were screened using the same process as above, and three additional articles were identified for inclusion in the review. Figure 1 highlights the Preferred Reporting Items for Systematic Reviews and Meta-Analyses (PRISMA) flow diagram.

**Table 1:** Inclusion and exclusion criteria *This criterion was removed for the second pass of the literature review. SCORAD: SCORing Atopic Dermatitis

Inclusion	Exclusion
*Subject age of 18 years or older	Systematic reviews/review articles
Randomized controlled trial	Published before 2007
Placebo	Receiving other treatments targeted at symptoms of atopic dermatitis
Use of SCORAD	
Human subjects	
Topical probiotic(s)	
English language	

Data Extraction

Data was extracted from the included articles by one reviewer (EF). Data extracted from each article included the probiotic(s) used, the study's duration, the days at which treatment effects were measured, and the age of the study subjects. Raw data of SCORAD values were not included in the Gueniche et al. study [15], so SCORAD values were obtained using the software ImageJ (National Institutes of Health, Maryland, United States) to estimate values reliably based on graphs provided by the paper [16]. 

Statistical Analysis

Meta-analyses were performed for three time periods: (1) baseline, (2) two weeks, and (3) four weeks. The intervals at which outcomes were measured varied slightly between studies. For our analysis, measurements taken on days 0 and 1 were grouped as "baseline." Days 14 and 15 were grouped as "two weeks," and days 28, 29, and 30 were grouped as "four weeks." Four studies were used for the baseline assessments, two for the two-week assessments, and three for the four-week assessments. The standardized difference in means was used as the effect size index. Because we assumed that the studies in each analysis represented a random sample from the universe of potential studies, we employed a random-effects model for each analysis, with each analysis being used to make an inference to the universe of potential studies. In support of our use of a random-effects model, we provide the following two statements from a book on meta-analysis: "When studies are pulled from the literature, a random-effects model should be used because common sense indicates that the true effect size varies across studies" [17] and "This model assumes that the studies in the analysis are representative of a universe of comparable studies and that the results of the analysis will be generalized to that universe" [17]. In addition, one of our goals was to create a prediction interval for each time period, and the creation of a prediction interval requires the use of a random-effects model. As indicated in another recently published book [18], "a prediction interval includes the true effect size for 95% of all populations in the universe." In this book, the importance of prediction intervals in meta-analyses is stressed [18], and an article is cited that makes a plea for routinely presenting prediction intervals in meta-analyses [19]; therefore, to help interpret the results of our meta-analysis, a prediction interval (which is based upon the random-effects model) was calculated for each time. The prediction interval reflects the variation in treatment effects over different settings, including what effect is to be expected in future patients [19]. (More accurately, in 95% of all meta-analyses, the mean effect size will fall within the confidence interval) [17]. As explained in this cited book, the 95% confidence interval for a fixed-effects model tells us that the mean prevalence in the set of studies falls within this range, and the 95% confidence interval for a random-effects model tells us that the mean in the universe of comparable populations falls within this range, whereas the 95% prediction interval tells us that the prevalence in any single population can be as low or as high as the lower limit and the upper limit of the interval [17].

For our meta-analysis, the standardized mean difference (SMD) for a fixed-effects model was calculated with Hedges' g statistic [20], and the heterogeneity statistic was used to calculate the summary SMD for a random-effects model [21]. If the value 0 is not within the 95% confidence interval, the SMD is statistically significant at the 5% level (p<0.05) [22]. Cohen's rule of thumb for the interpretation of the SMD statistic is as follows: a value of 0.2 indicates a small effect, a value of 0.5 indicates a medium effect, and a value of 0.8 or larger indicates a large effect [22].

Alpha was set at 0.05; however, Amrhein et al. emphasized that the clinical importance of findings, not their statistical significance, be emphasized [23], and for the interpretation of results of our meta-analysis, the clinical importance of findings is emphasized. For our meta-analysis, the SMD for a fixed-effects model was calculated with Hedges' g statistic [20], and the heterogeneity statistic was used to calculate the summary SMD for a random-effects model [21]. If the value 0 is not within the 95% confidence interval, the SMD is statistically significant at the 5% level (p<0.05) [23]. Cohen's rule of thumb for the interpretation of the SMD statistic is as follows: a value of 0.2 indicates a small effect, a value of 0.5 indicates a medium effect, and a value of 0.8 or larger indicates a large effect [22]." We performed our meta-analysis with Comprehensive Meta-Analysis Version 4 [24].

Results

Our search strategy yielded four articles that met the inclusion criteria [15,25-27] (Table 2). Two of these studies used the same bacterium as a probiotic, *Vitreoscilla filiformis *[15,27]. One study used *Lactobacillus reuteri*, and one used a mix of *Bifidobacterium *and *Lactobacillus *species [25,26]. Articles tested the use of probiotics in treating AD, although different timelines were used for testing. 

**Table 2:** Summary of all included articles SCORAD: SCORing Atopic Dermatitis; TEWL: transepidermal water loss

Study	Probiotic	Duration of study/days SCORAD measured	n/age range of subjects in years (placebo, treatment)	Percent change from baseline in SCORAD	Additional findings
Noll et al., 2021 [25]	Mixture (*Bifidobacterium breve, Bifidobacterium animalis lactis, Lactobacillus casei, L. gasseri, L. plantarum, L. rhamnosus*)	2 weeks/0, 9, 11, 14	7, 7/5+	Synbiotic baths: -40.7%, -49.7%, and -56.8% on days 9, 11, and 14, respectively. Placebo: -2.3%, +1.3%, and +7.1% on days 9, 11, and 14, respectively	Analysis of skin flora showed successful colonization with bacteria from the probiotic treatment, but the abundance of *Staphylococcus aureus* did not significantly change. Subjects in the probiotic group subjectively reported less pruritis and less dryness at the end of the study compared to the control group
Butler et al., 2020 [26]	*Lactobacillus reuteri* DSM17938	8 weeks/0, 28, 56	17, 17/19-66, 19-57	Probiotic ointment: -27.7% and -45.7% on days 28 and 56, respectively. Placebo: -31.9% and -41.0% on days 28 and 56, respectively	Improvement of skin dryness in the probiotic group was the most pronounced change during the study
Gueniche et al., 2008 [15]	Vitreoscilla filiformis	4 weeks/0, 15, 29	25, 26/6-70, 6-70	Probiotic cream: -29.7% and -56.1% on days 15 and 29, respectively. Placebo: -7.8% and -16.3% on days 15 and 29, respectively	The probiotic group showed a significantly greater reduction in SCORAD when compared to the control at days 15 and 29. Skin barrier function was measured using the biomarker TEWL. There was a significant reduction in TEWL in both groups from their baseline values at the end of the study, but there was no significant difference between the probiotic and control. The group treated with probiotics had decreased the colonization of *Staphylococcus aureus *and *Escherichia coli* compared to the control at the end of the study, but the finding was not statistically significant
Seite et al., 2017 [27]	*Vitreoscilla filiformis* (LRP-VFB)	4 weeks/1, 28	22, 24/0-63, 0-63	Probiotic cream: -15.5% on day 28. Placebo: +32.0% on day 28	The probiotic group had significantly increased levels of *Xanthomonas *at the end of the study compared to the control. Colonization with *Staphylococcus aureus* increased in the control group, but not in the treatment group. The difference was not statistically significant

SCORAD

**Figure 2:**
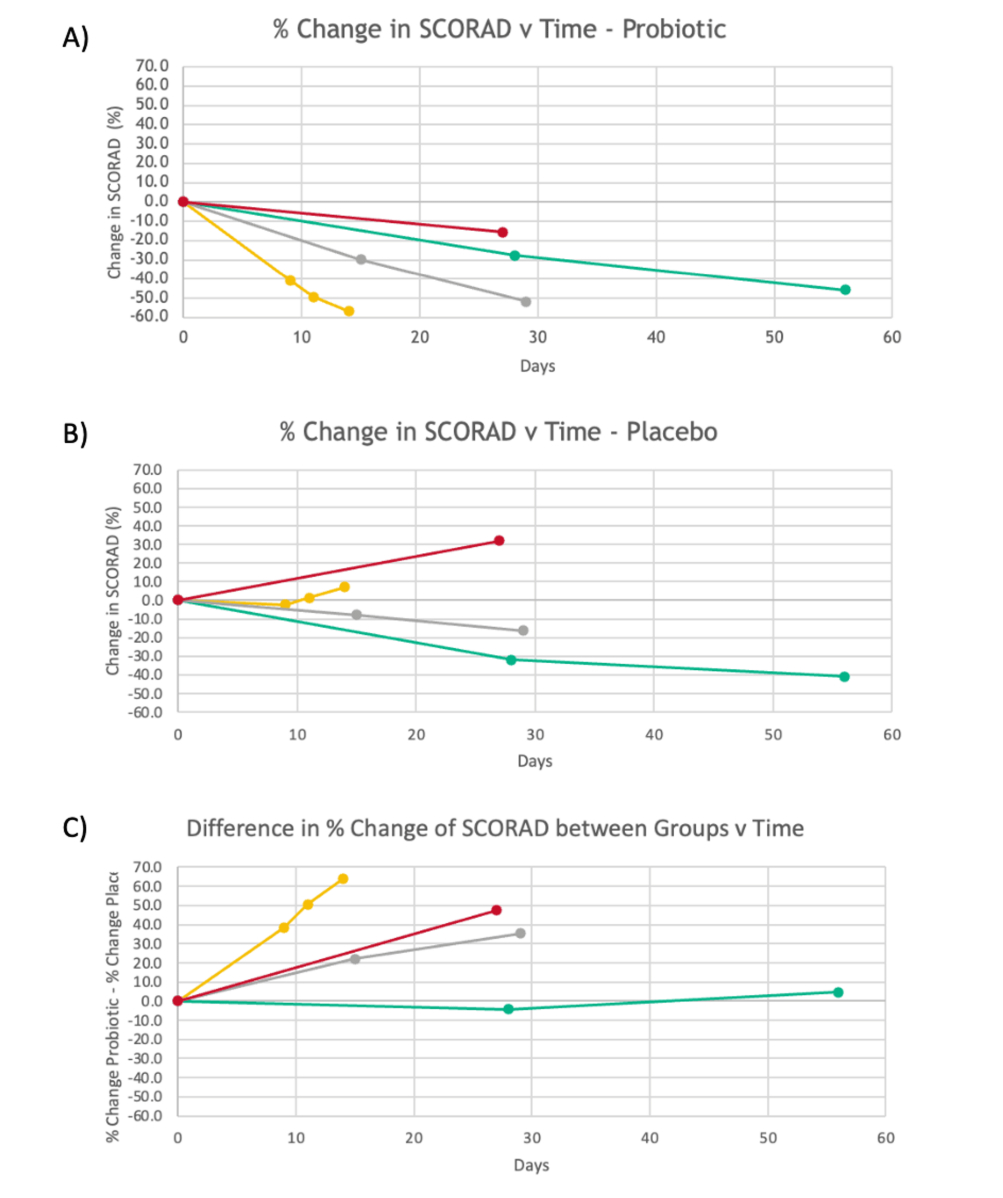
Percent change from baseline in SCORAD vs time (A) Percent change in SCORAD vs time for probiotics for all studies. (B) Percent change in SCORAD vs time for placebo for all studies. (C) Difference in percent change in SCORAD between groups vs time for all studies. Yellow lines indicate Noll et al. [25], green lines indicate Butler et al. [26], grey lines indicate Gueniche et al. [15], and red lines indicate Seite et al. [27]. SCORAD: SCORing Atopic Dermatitis

SCORAD is a standardized scoring tool to rate the severity of AD by physician and patient ratings of pruritis, dryness, and redness. Higher SCORAD values indicate more severe symptoms, whereas a low SCORAD indicates milder disease severity. In the articles that were included in our analysis, the percent change in SCORAD demonstrated reduced eczema severity with the use of probiotics in all four studies (Figure 2A), whereas placebos only reduced eczema severity in two of the four studies (Figure 2B). The percent change in SCORAD from the probiotic groups was subtracted from the percent change in placebo at each respective time point and graphed; positive values indicate that the probiotics outperformed the placebo at that charted point (Figure 2C). This figure demonstrates how probiotics outperformed placebos across all studies, except for the Butler et al. study at 28 days [26]. It should be noted, however, that the Butler et al. study showed a benefit from the probiotics compared to placebo later in the study at 56 days. 

Meta-Analysis

**Figure 3:**
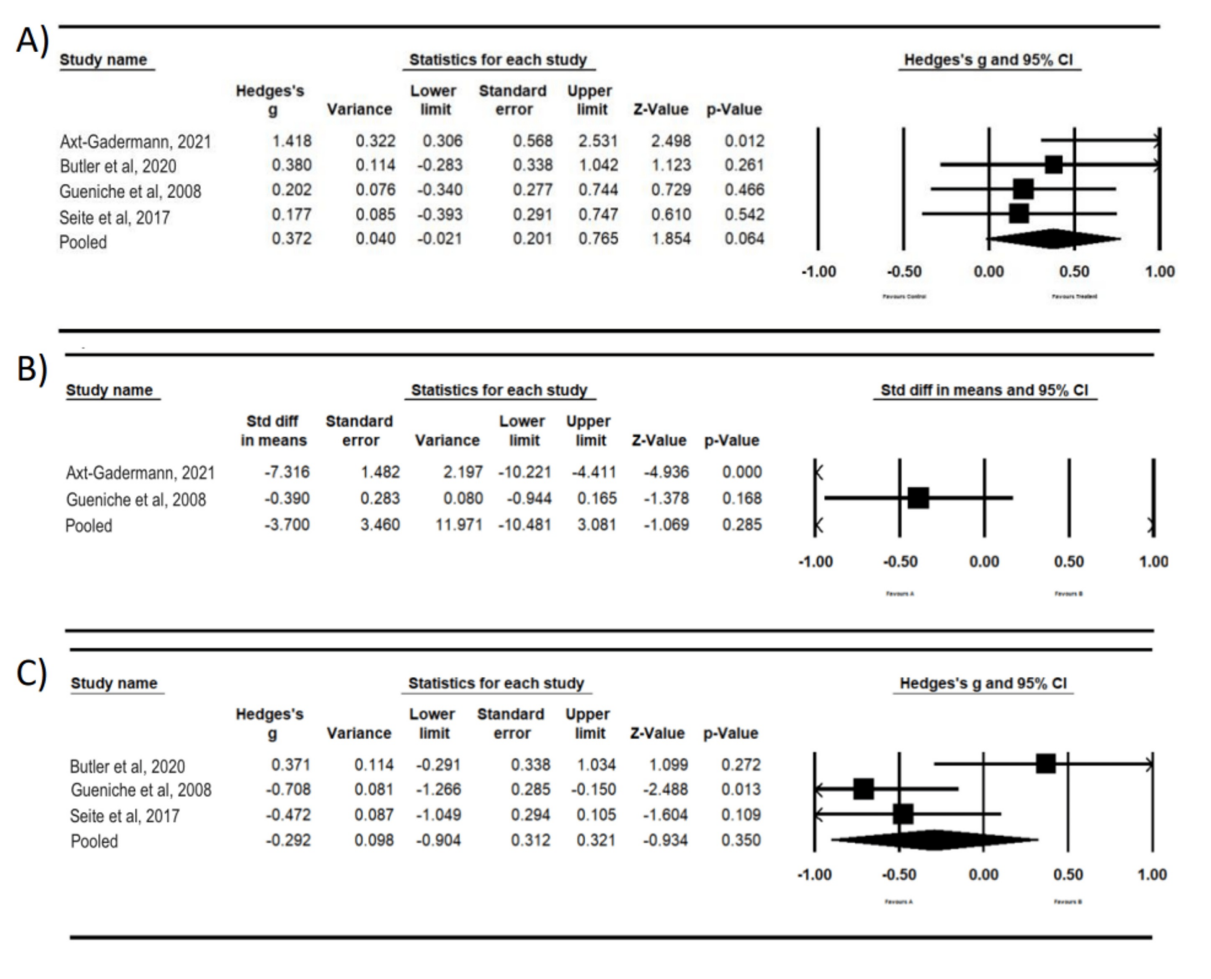
Summary values for dermatitis (A) Baseline values for four studies and the pooled values for the random-effects model. (B) Two-week values for two studies and the pooled values for the random-effects model. (C) Four-week values for four studies and the pooled values for the random-effects model.

Meta-analyses were performed for the placebo and probiotic treatment groups. For the baseline data, the mean effect size is 0.372 with a 95% confidence interval of -0.021 to 0.765 (Figure 3A). The mean effect size in the universe of comparable studies could fall anywhere in this interval. The Z-value tests the null hypothesis that the mean effect size is 0. The Z-value is 1.854 with p=0.064. Using a criterion alpha of 0.050, we cannot reject this null hypothesis of no effect. If the true effects are normally distributed (in g units), an estimate of the prediction interval is -0.889 to 1.633 (Figure 3A). The true effect size in 95% of all comparable populations falls in this interval.

**Figure 4:**
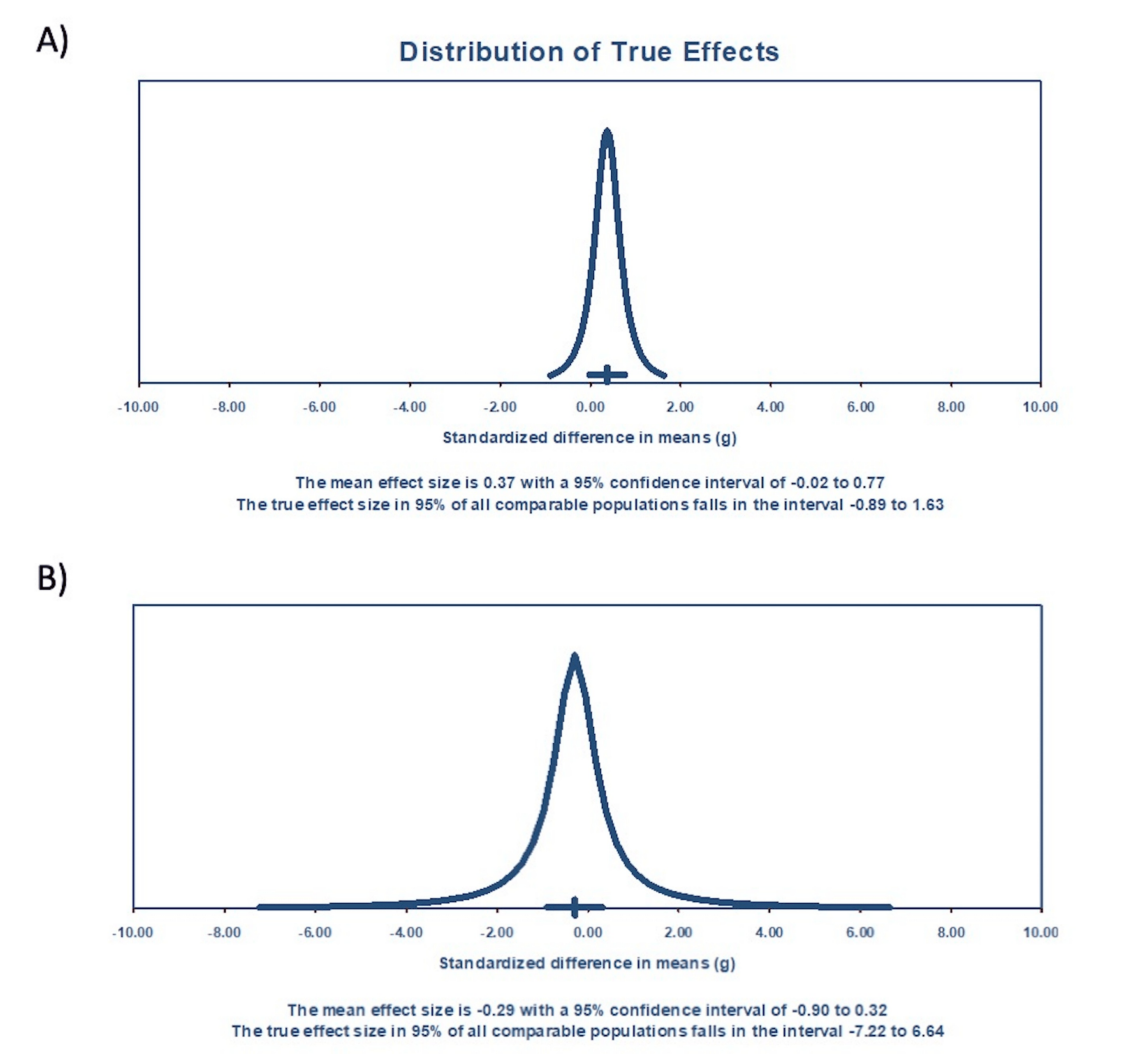
Prediction intervals for dermatitis at baseline and at four weeks A two-week prediction model could not be created because a minimum sample size of 3 is required. (A) Prediction interval for baseline values. (B) Prediction interval for four-week values.

These calculations for baseline data included only four studies. It has been suggested that 10 studies are a useful minimum for a meta-analysis 15. Therefore, estimates of heterogeneity based on less than 10 studies may not be reliable. A prediction interval and other functions could not be performed for the two-week data because a minimum sample size of 3 is required, and for the two-week assessments, there were only two studies. For the two-week data, the mean effect size is -3.700 with a 95% confidence interval of -10.481 to 3.0815 (Figure 3B). The mean effect size in the universe of comparable studies could fall anywhere in this interval. The Z-value is -1.069 with p=0.285, which does not reject the null hypothesis of no effect. For the four-week data, the mean effect size for the three studies is -0.292 with a 95% confidence interval of -0.904 to 0.321 (Figure 3C). The mean effect size in the universe of comparable studies could fall anywhere in this interval. The Z-value is -0.934 with p=0.350. Using a criterion alpha of 0.050, we cannot reject this null hypothesis of no effect. An estimate of the prediction interval is -7.221 to 6.637 (Figure 4B). The true effect size in 95% of all comparable populations falls in this interval. These calculations for four-week data included only three studies; therefore, heterogeneity estimates may not be reliable.

To summarize the results of our assessments, none of our meta-analyses rejected the null hypothesis of no effect (p≥0.064). The prediction interval at baseline indicated that with treatment, many future patients would experience benefits; however, at four weeks, the prediction interval indicated that it would be essentially a coin toss as to whether a future patient would experience a benefit. A limitation of our assessments is that our sample sizes for baseline, two weeks, and four weeks were small, respectively, four studies, two studies, and three studies.

Synthesis

AD lesions show an increased abundance of *S. aureus* and reduced diversity of the skin microbiome [5,7]. The most common treatments for AD include topical emollients and topical glucocorticoids, and many additional medications are available, including phototherapy, monoclonal antibodies, and systemic glucocorticoids. Many patients have AD that is resistant to treatment, leading to many clinical research studies testing probiotics. We performed a systematic review and meta-analysis of studies that investigated the use of topically applied probiotics for AD treatment. Overall, only a few studies met our inclusion criteria. The included studies demonstrate the benefit of probiotic application in treating AD as measured by a standard scoring system (SCORAD); however, that benefit varied at the different time points measured. Three of the studies included microbiome sampling of the patients in these studies. Two of the studies showed a decrease in *S. aureus* presence [15,27], but one study showed that *S. aureus* presence remained stable [25]. Interestingly, the studies that reduced *S. aureus* were the studies that used *V. filiformis*, which, as noted below, was the most effective treatment used.

Initially, this study was to be a systematic review of AD in adults, but there were not enough studies to carry out that systematic review. We modified the criteria to accept studies that included both children and adults. Even so, only four studies met the criteria. We believe that this is of interest to the research community, there seems to be a large number of studies about probiotics in the treatment of AD, but they are almost entirely oral probiotics. This review and meta-analysis reports the effectiveness of topical probiotic efficacy in treating AD in clinical trials that include adults. A major challenge was that studies used different probiotic formulations and different study periods. The meta-analysis of all the studies showed a beneficial effect on reducing AD symptoms at two weeks but not at four weeks. The Butler et al. [26] study showed no effect at four weeks, but showed a beneficial effect at eight weeks, suggesting that topical probiotics are effective, but more work needs to be done to determine the length of time probiotics should be administered for each probiotic formulation. Until more clinical trials are performed using standardized treatments and treatment lengths, it will be challenging to determine the effectiveness of topical probiotics. The one study that showed little effect on SCORAD for up to four weeks used a probiotic with only a single organism, *L. reuteri* [26]. Two of the other studies used *V. filiformis*, and one study used a symbiotic, a mix of several bacterial species, along with a prebiotic (Table 1). Future studies should focus on probiotics that show effectiveness, and while there are only two studies, *V. filiformis* is promising as a probiotic treatment for AD [15,27]. 

While AD commonly affects infants and young children, adults are affected as well, with as many as 10% of adults reporting eczema [28,29]. The gut microbiome has been linked with skin health and may be important in preventing inflammatory processes associated with AD by stimulating anti-inflammatory cytokines and inducing the production of regulatory T cells [30,31]. Commensal microbiota may interfere with *S. aureus* through the production of antimicrobial peptides [32], by preventing biofilm formation [33], and by influencing the immune system to produce TLRs promoting pathogen clearance [34]. Two of the clinical trials used *Lactobacilli*, bacteria that have been shown to produce bacteriocins [25]. *V. filiformis* has been shown to stimulate the innate immune system and activated TLR2 [27]. These and other probiotics may help restore skin integrity and reduce the amount of *S. aureus* present. Decreasing *S. aureus* growth on the skin could increase diversity and reduce inflammation. Clinical trials using oral probiotics to modify the gut microbiome have been performed, but in adults, oral probiotics have shown mixed effects [32,35]. Our systematic review evaluated topical probiotics to determine whether there was an effective treatment for adults.

A recent systematic review analyzed many AD treatments, including oral and topical probiotics, topical emollients, biologics, pharmaceuticals, and many other therapies [32]. They found that treatments across the spectrum had positive effects on AD outcomes. Oral probiotics generally improved AD severity in children and adults, although some studies in children showed no effect. They found that topical probiotics generally improved AD, but they also included non-clinical trials in their results. They also found that there were significant benefits from biologics. Our systematic review and meta-analysis used only double-blind, randomized clinical trials, and all studies we analyzed included adults. We found that while there was a general reduction in SCORAD from topical probiotic use in three out of four studies, we could not reject the null hypothesis that the treatment had no effect at four weeks. This is consistent with other research. Greenzaid et al. found that most of the reported studies show a positive effect on SCORAD or other indicators of AD, but there were also studies showing no effect [32]. Many treatment studies show a benefit to AD patients, but the mixed results show that there is a need to determine which treatments work, why they work, and the appropriate length of treatment. In this systematic review, the *V. filiformis* probiotics using a cream as a vehicle produced the best effects. Interestingly, *V. filiformis* was originally isolated from water in hot springs, and *V. filiformis* extracts are used in cosmetics. These cosmetics have been shown to decrease inflammation and maintain the skin barrier, properties consistent with relieving AD symptoms [36]. While these treatments produced positive results, there are many possible probiotic bacteria and delivery methods. Additional studies are still needed to determine the most effective treatments.

An advantage of topical probiotic treatment is that the immune system in the skin is dynamic, responding quickly to changes. Altering the bacteria that are present may rapidly change the immune signaling. Using topical probiotics in conjunction with other treatments may lead to quicker and more beneficial outcomes. All four clinical trials that met the criteria, using topical probiotics in randomized, double-blind studies, showed positive effects over the length of the trial. Our meta-analysis of all probiotic formulations over time showed that this treatment is only effective over the first two weeks of treatment, but there is no clear effect at longer time points. This suggests only a short-term effect for these treatments. Because endpoints varied between studies, the longest common endpoint, four weeks, was analyzed and showed no significant effect. We recommend that additional clinical trials be performed using the treatments that were clearly successful, particularly those using *V. filiformis*, using more patients over a longer period to determine treatments that will be effective enough for widespread use.

## Conclusions

We investigated whether topical probiotics were effective in alleviating AD in adult populations in clinical trials. We focused on topical probiotics rather than oral probiotics, because there has been a research focus on oral probiotics, particularly in children, but fewer studies on topical probiotics. While we found many published studies testing the effectiveness of probiotics on AD in animal and human studies, there were four that used topical probiotics in placebo-controlled, double-blind clinical trials with adult patients. While all four studies showed that topical probiotics significantly alleviated AD symptoms, a meta-analysis using the data from all these studies showed no benefit. Interestingly, the two studies that used *V. filiformis* in cream/emollient vehicle showed great benefit in reducing AD symptoms as measured by improved SCORAD measurements. While *V. filiformis* produced the best outcomes, the other probiotic formulations individually showed significant benefits to patients. Due to the low number of studies that have been reported, there is a need for more topical probiotic studies to be performed. While *V. filiformis* topical probiotic treatment seems promising, there is not enough data to rule out other topical treatments.
